# High-order asymptotic methods provide accurate, analytic solutions to intractable potential problems

**DOI:** 10.1038/s41598-024-54377-2

**Published:** 2024-02-20

**Authors:** Alexander W. Wray, Madeleine R. Moore

**Affiliations:** 1 Department of Mathematics and Statistics, University of Strathclyde, Livingstone Tower, 26 Richmond Street, Glasgow, G1 1XH UK; 2 Department of Mathematics, School of Natural Sciences, University of Hull, Cottingham Road, Hull, HU6 7RX UK

**Keywords:** Potential problems, Asymptotic methods, Electrostatics, Evaporation, Fluid dynamics, Applied mathematics, Electrical and electronic engineering, Fluid dynamics, Applied mathematics, Electrical and electronic engineering

## Abstract

The classical problem of determining the density and capacity of arrays of potential sources is studied. This corresponds to a wide variety of physical problems such as electrostatic capacitance, stress in elastostatics and the evaporation of fluid droplets. An asymptotic solution is derived that is shown to give excellent accuracy for arbitrary arrays of sources with non-circular footprints, including polygonal footprints. The solution is extensively validated against both experimental and numerical results. We illustrate the power of the solution by showcasing a variety of newly accessible classical problems that may be solved in a rapid, accurate manner.

## Introduction

**Figure 1:**
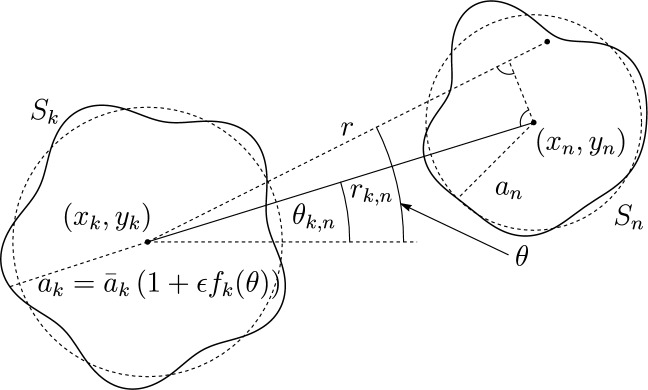
Schematic showing notation used in the mathematical description. Two sources occupy $S_i$, $i = k, n$, with boundaries denoted by $a_i$. The source centres are denoted by $(x_i,y_i)$ and polar coordinates $(r,\theta )$ are defined in relation to the centre of $S_k$. The distance and angle between the centres are denoted by $r_{k,n},\theta _{k,n}$, respectively.

We examine the classical mathematical problem of solving the three-dimensional Laplacian mixed boundary value problem

1 $$\begin{aligned} \nabla ^2 c=0, \, \lim _{|\textbf{x}|\rightarrow \infty }c=0,\, {\left\{ \begin{array}{ll} c|_{z=0}=c_S(x,y),&{}\quad \hspace{-0.3cm}(x,y)\in S\\ c_z|_{z=0}=0,&{}\quad \hspace{-0.3cm}(x,y)\notin S \end{array}\right. }, \end{aligned}$$

where the *potential* $c_S(x,y)$ is specified, and $S=\cup _k S_k$ is a domain in the $z=0$ plane, where the $S_k$ are flat, disjoint, simply-connected components of the domain, termed the *sources*, as shown in Fig. 1. The problem is to determine the *density* $\sigma _k$ and *capacity* $C_k$ for each source,

2 $$\begin{aligned} \sigma _k=-\frac{\partial c}{\partial z} \; \text{ for } \; (x,y)\in S_k, \quad C_k=\iint \limits _{S_k} \sigma _k\, \text{d}A. \end{aligned}$$

Suitably dimensionalized, the density corresponds to a variety of physical situations (see Chapter 1 of Ref.^1^ and the many references therein) including heat flux in thermostatics^2,3^; charge density in low conductivity electrostatics and charge flux in high conductivity electrostatics^4^; diffusive liquid flux through a membrane^5^; evaporative flux of droplets^6^; imbibition flux into porous layers^7^; transmission in magnetic polarizability^8^; the stress imposed by stamps on linear elastic media^9^; diffusion across the surface of nanobubbles and nanodroplets^10^; and the growth of ice crystals^11^ among other applications.

Accordingly, the problem has been much studied: the first solution for a single, circular source was given by Weber in 1873^12^, and it has since been the subject of major books^1,13^ and reviews^6,14^. However, despite over 150 years of attention, few exact closed-form solutions are known: only those for a circular disk^12,15^ and an ellipse^16^ (and solutions for non-flat sources^17–19^). There have been attempts to provide solutions for more general planar shapes, including approximate formulations^20^, and asymptotic solutions for monochromatic source boundaries^21^. In addition, solutions have been determined for both the density and capacity for arrays of multiple circular sources^22–24^.

Unfortunately, in practice, sources typically have footprints with complex polychromatic shapes, and rarely occur in isolation. This has, to date, meant that Eq. (1) must be tackled using time-consuming numerical methodologies such as the boundary element method (BEM)^25^ or the finite element method (FEM)^21,26^. Unfortunately, the singular behaviour of the density at the boundaries $\partial S_k$ of the sources has meant that in even the simple case of two circular sources, FEM was not reliably accurate enough to assess the accuracy of analytical results^22^, significantly limiting the scale of accessible systems.

Here, we present a novel solution to this issue by determining an explicit solution for general arrays of sources with star-convex domains. While formally only valid close to circularity, the solutions are tested extensively against both experiments and numerical computations to demonstrate validity even far from this limit. As representative examples, we demonstrate that this can for the first time provide accurate solutions to long-standing problems in electrostatics, droplet evaporation and printing problems among others.

## Results

As shown in Fig. 1, we consider *N* sources with centres $(x_k, y_k)$ and domains $S_k$ with boundaries $r=a_k(\theta )={\bar{a}}_k(1+\epsilon f_k(\theta ))$ in the plane $z=0$, where $(r,\theta )$ are polar coordinates relative to the centre of source *k*; ${\bar{a}}_{k}$ is the average contact radius of droplet *k* normalized by the average contact radius of droplet $k = 1$; and $0<\epsilon \ll 1$. Note that hence ${\bar{a}}_1 = 1$. While in the problem of an isolated source we present results for the case $k=1$, accommodating ${\bar{a}}_{k}\ne 1$ is a simple matter of rescaling. The distance between the centres of sources *k* and *n* is $r_{k,n}$.

The potential $c(r,\theta ,z)$ satisfies the potential problem Eq. (1) in the upper half plane. This problem may be recast in a Green’s function formulation and we may then exploit the smallness of the parameter $\epsilon$ to find an asymptotic solution for the density and capacity. This procedure is described in the Supplementary Information (SI). While trivially generalisable, the solution for the density when $c_S\equiv 1$ is

3 $$\begin{aligned} \sigma _i=\sigma _i^I\left[ 1-\sum _{j\ne i} S(r,\theta ;i,j)\right] , \end{aligned}$$

where $\sigma _i^I$ is the density of source *i* in isolation (see Eqs. (9), (11), (20), (30) in the SI), and $S(r,\theta ;i,j)$ is the shielding of source *i* due to source *j* (see Eq. (38) in the SI).

Note that the capacity $C_i$ in the presence of other sources, which is required to evaluate *S* and hence $\sigma _i$, may be determined from

4 $$\begin{aligned} C_i^I=C_i+\sum _{j=1,j\ne i}^N \frac{2}{\pi }\arcsin \left( \frac{a_i}{r_{i,j}} \right) C_j, \end{aligned}$$

as shown by Ref.^24^, where $C_i^I$ is the capacity of source *i* in isolation, determined from $\sigma _i^I$.

In principle, the solutions given by Eqs. (3) and (4) are only valid for large $r_{k,n}$ and small $\epsilon$, that is, when the sources are far apart and almost circular. However, as we shall demonstrate below, the solutions prove robust even when both are of order unity.

In the following suite of example applications, where possible we compare against exact or experimental results. Where numerical validation is required, we solve the problem using COMSOL^27^, as described by Ref.^21^.

## Single source problems

We shall first illustrate the veracity and strength of Eqs. (3) and (4) for a single source. In the interests of brevity, all suffices denoting source number are suppressed in this section.

### Capacity of an isolated source

The capacity of an isolated source is a common quantity of interest in electrostatics, where the source is an electrode termed the capacitor, the density is the charge distribution, and the capacity is the electrostatic capacitance: the total charge on the capacitor^4^. Capacitors are ubiquitous in modern electronics due to their utility in contexts including power conditioning and energy storage^28^.

Unfortunately, as capacitance must be determined by solving a problem of the form Eq. (1), it suffers the aforementioned lack of analytic solutions. This has led to significant effort, both experimental^29^ and numerical^9,30–33^, to determine the capacitance of various shapes of electrode. Even approximate solutions for the simple case of a square electrode are still an ongoing area of interest^34^.

To this end, as we shall demonstrate, the asymptotic solution Eqs. (3) and (4) represents a step change in the accuracy of analytic expressions for the capacitance. In order to quantify the accuracy of the capacitance predicted by Eqs. (3) and (4), a dimensionless coefficient *g* depending only on the shape of the source is defined via

5 $$\begin{aligned} 1=\frac{C}{2\pi g \sqrt{A}}, \end{aligned}$$

where *A* is the area of the domain^20,34^. For a square electrode, Ref.^29^ gives an experimental value of $g=0.37532$, COMSOL gives a numerical value of $g=0.368$, and several approximate values exist in the literature: Ref.^34^ offers $g=0.36 \pm 0.01$, Ref.^20^ offers $g=0.3612$, Ref.^32^ offers $g=0.3363$ and Ref.^30^ offers $g=0.3613$. Our new solution Eq. (3) gives $g=0.367$, which lies within the bounds prescribed by Ref.^34^ and is closest to the COMSOL result (an error of 0.25%). All these values differ somewhat from the experimental value of Ref.^29^, likely due to a combination of finite electrode thickness effects and experimental measurement error. In particular, the 2% error between COMSOL and experiment is therefore a threshold below which a model might reasonably be considered exact for practical purposes.

**Figure 2:**
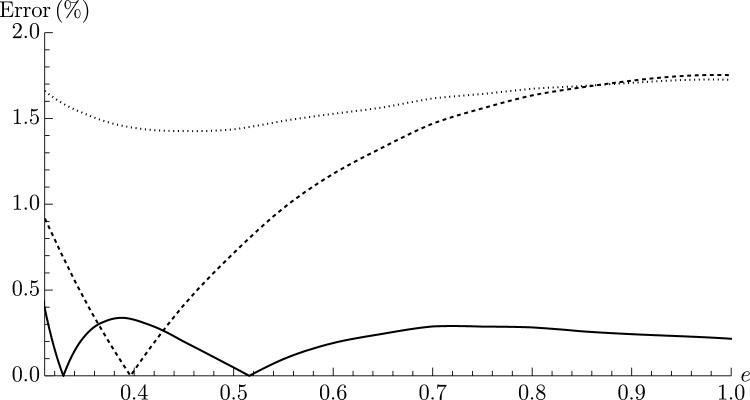
Absolute relative error in the capacitance for a rectangular electrode of eccentricity *e*, according to Eq. (3) (solid line), Fabrikant’s approximation^20^ (dashed line) and Solomon’s approximation^30^ (dotted line) compared to numerical results from COMSOL. Notably, the two approximations from the literature are specifically derived for rectangular contact lines, yet perform substantially worse than Eqs. (3) and (4).

In applications, electrodes are often rectangular rather than square. Therefore, in Fig. 2 we show the absolute relative error of Eqs. (3) and (4) and the approximate expressions of Refs.^20,30^ compared to COMSOL for the coefficient *g*, for varying eccentricities *e* of rectangle. We find Eqs. (3) and (4) give excellent agreement, surpassing the accuracy even of models designed specifically to accommodate rectangular geometries^30^.

This provides a stringent test of Eqs. (3) and (4) due to the presence of sharp corners, and increasing deviation from circularity with eccentricity—other tests of footprints that are smoother and/or closer to circularity find, as anticipated, even better agreement with the exact solutions.

**Figure 3:**
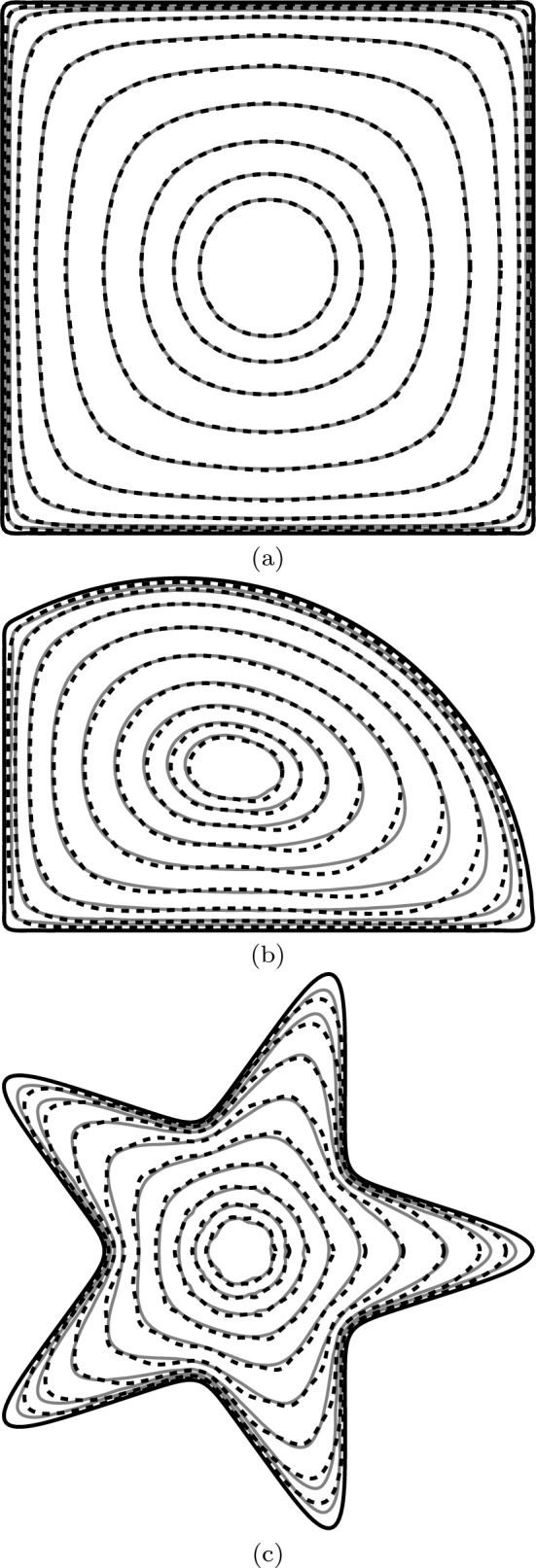
Density as predicted by asymptotics (dashed lines) and COMSOL (solid lines) from (**a**) a square domain ($g=0.368$; 0.25% error); (**b**) an irregular domain ($g=0.371$; 0.977% error); (**c**) a star-like domain ($g=0.407$; 0.76% error).

### Density of an isolated source

In many contexts, the spatial variation of the density is crucial. For example, an accurate expression for the density affords a rapid way of computing the potential anywhere in space via the Green’s function formulation of Eq. (1). Densities for several shapes are given in Fig. 3. These examples have deliberately been selected as ones that test the limits of Eq. (3) due to their significant non-circularity and sharp corners. We see strong agreement in the density contours for each shape (alongside $<1\%$ error in *g* in each case). Shapes with less acute corners, such as pentagons, give even better results, and corresponding plots yield results for which COMSOL and Eq. (3) are indistinguishable.

Of particular note in Fig. 3 is the change in shape of the density contours as we move from the edge of the source towards its centre. Near the boundary, the contours are roughly the same shape as the boundary itself, while near the centre, the contours are closer to circular or elliptical. This is in stark contrast to the approximate solution of Ref.^20^, which gives contours that are always scaled versions of the boundary and illustrates the necessity of the higher-order corrections in the solution Eqs. (3) and (4) presented herein. A similar finding for a source with a monochromatic boundary was discussed in Ref.^21^.

In fact, our explicit solution allows for a deeper investigation of the behaviour of the shape of contours close to the centre of the source. Suppose that the contact line profile has the Fourier series

6 $$\begin{aligned} f(\theta ) = \sum _{j=2}^{M}B_{j}(\theta ), \quad B_{j}(\theta ) = a_j\cos (j\theta ) + b_j\sin (j\theta ), \end{aligned}$$

where $M\ge 2$, and the $j = 1$ term may be eliminated by a suitable choice of coordinate origin within the source. Then, an asymptotic expansion as $\epsilon , r\rightarrow 0$ of the density of an isolated source, $\sigma _I$, as given by Eqs. (9), (11) and (20) in the SI gives

7 $$\begin{aligned} \sigma = \frac{2}{\pi }\left( 1 + \frac{r^2}{2} -\epsilon B_2(\theta ) + O(\epsilon ^2,r^3)\right) \quad \text{ as } \quad r,\epsilon \rightarrow 0. \end{aligned}$$

Thus, we see that if the contact line geometry is such that the coefficients $a_2$ or $b_2$ in the Fourier series Eq. (6) are nonzero, then the contours of the density exhibit $O(\epsilon )$ perturbations from circularity, and thus appear elliptical close to the origin. Otherwise, they are circular up to $O(\epsilon ^2)$.

#### Application to the coffee-ring effect in evaporating droplets

As a motivating example of the utility of the density, we examine the coffee-ring effect in thin, non-circular, solute-laden droplets. Due to applications varying from microscale patterning to DNA mapping to blood spatter analysis^35,36^, this field has attracted significant experimental and numerical attention^37–42^. However, most previous analytical attempts were confounded by the absence of a suitable expression for the evaporative flux (i.e. the density) and have thus concentrated on simple geometries such as axisymmetric or elliptical droplet footprints. The existence of the asymptotic solution Eq. (3) now opens up a new approach for these more complex problems.

We follow the formulation of Ref.^23^, whereby the solute is dilute so that transport occurs purely via advection. For thin droplets, the depth-averaged flow velocity ${\bar{u}}$, the liquid pressure *p* and the droplet free surface *h* are given by

8 $$\begin{aligned} {\bar{u}}&= -\frac{H^2}{3\alpha (1-t/t_f)}\nabla P, \nonumber \\ p&= -\frac{1}{\alpha ^3(1-t/t_f)^3}P, \nonumber \\ h&= \alpha (1-t/t_f)H, \end{aligned}$$

where $\alpha =1/\iint H \,\text{d}A$ and the extinction time of the droplet is $t_f = 1/\iint \sigma \,\text{d}A$. Driven by the dominance of capillarity, the scaled free surface profile $H(r,\theta )$ satisfies the Young-Laplace equation

9 $$\begin{aligned} \nabla ^2H=-1, \quad \left. H\right| _{r = a(\theta )} = 0, \end{aligned}$$

with suitable regularity conditions at the origin. The scaled pressure may be determined from the thin film equation

10 $$\begin{aligned} \nabla \cdot (H^3 \nabla P) = \sigma -(\alpha /t_f) H, \quad \left. H^3 \nabla P\cdot \textbf{n}\right| _{r = a(\theta )} = 0, \end{aligned}$$

where $\textbf{n}$ is the outward-pointing normal to the pinned contact line.

Ignoring all effects of finite particle size and jamming, the streamlines are time-independent in the droplet and hence coincident with the pathlines^43^. Hence, once the liquid has fully evaporated, the mass $M(\theta _c)$ of residue accumulated at the contact line between $\theta =0$ and $\theta =\theta _c$ is the total initial solute located between the streamlines originating from the stagnation point within the droplet and finishing at $\theta =0$ and $\theta =\theta _c$. The solute residue density may then be found by evaluating $D=\text{d}M/\text{d}s$, where *s* is the arc length around the contact line^23^. This may be used as a measure of the strength of the coffee-ring effect. While the residue density can be computed analytically given a suitable asymptotic expression for *H*, in order to test the behaviour of Eq. (3) in isolation, *H* is computed numerically, and determination of the residue density reduces to simple quadrature.

As a stringent test of our asymptotic solution, we compare predictions of the residue density with experimental deposit results from Fig. 3b in Ref.^41^, which considers a droplet of coffee with a triangular contact line. In particular, the fact that Fig. 3b of Ref.^41^ does not exhibit saturation at any point (i.e. the pixels never have a lightness of exactly zero, indicating non-zero transmittance throughout the footprint of the droplet) means that the density can be determined via the Beer–Lambert law^21^. To minimise experimental noise, the extracted data is averaged with itself rotated by $2\pi /3$ and $4\pi /3$, as well as the reflection thereof. Note that, as the total mass of residue is unknown, this introduces one multiplicative fitting factor. This is chosen to scale the total residue in Ref.^41^ to coincide with the total residue in the analytical predictions.

**Figure 4:**
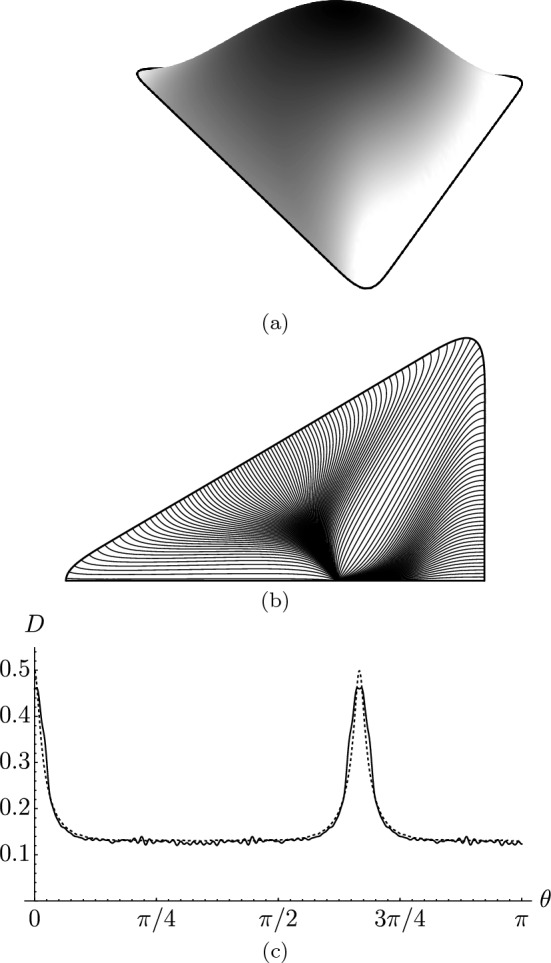
Deposition from a triangular droplet. (**a**) The numerically-calculated droplet profile. (**b**) The flow streamlines found using Eq. (3). (**c**) The residue density *D* as a function of polar angle around the contact line from the model (dashed line) and experimental data from Fig. 3b in Ref.^41^ (solid line) extracted using the methodology described in the main text.

We now compare this data to the thin-droplet model with our asymptotic solution for $\sigma$. The droplet free surface profile is found by solving Eq. (9) numerically and is shown in Fig. 4a. With this in hand, the streamlines may be calculated from Eqs. (8) and (10), and are shown in Fig. 4b. Finally, we may use calculated streamlines to calculate the residue density *D* given in Fig. 4c alongside the experimental data. The agreement is remarkably good, especially considering that our solution is based on a nearly-circular assumption and, moreover, the model does not take into account solutal diffusion^44,45^, jamming^18^, or the finite contact angle^46^, which may increase accuracy even further.

## Multiple source problems

### Capacities of multiple sources

In many industrial applications, it is desirable to examine the evaporation rate of extremely large arrays of sources^47–50^. For small arrays (*O*(10) sources), we may realistically use FEM packages such as COMSOL. For larger arrays this approach rapidly becomes impractical due to prohibitively slow speeds and infeasible memory requirements. This can be alleviated by using accurate reduced-order models such as Eq. (4), which when solved directly can comfortably handle $O(10^4)$ sources.

However, industrial problems can be many orders of magnitude larger. We examine the illustrative example of the manufacture of OLED screens in which the sources are printed pixels, the evaporation of which is both the rate-limiting manufacturing step and the critical factor determining defect rate^51^. What is more, ultra-high definition screens have $O(10^8)$ pixels^52^. It is therefore instructive to determine an efficient way to solve Eq. (4) for large arrays.

For very large arrays, a natural approach is to neglect edge effects as a first approach and model the system as appearing locally like a periodic array. However, this formulation presents a mathematical problem as it does not converge: neighbouring pixels decrease in effect as 1/*r* but increase in number as $r^2$. In fact, this fails even for an infinite line of pixels, analogous to the two-dimensional single source problem^53^.

While the problem could be approached using a Barnes–Hut formulation^54^, here we derive a more straightforward methodology. Although our approach can readily be extended to more general configurations, as an illustration, we will consider a simpler model problem, namely a square array of circular pixels of unit radius located at

11 $$\begin{aligned} (x_i,y_j) = \left( i\times d,j\times d\right) , \quad \text{ for } \quad {i,j}\in [-N,\ldots ,N]. \end{aligned}$$

The capacity of each pixel is denoted by $C_{i,j}$. We pass to a continuum formulation by considering a function $C(x,y)$ coinciding with $C_{i,j}$ at $C(x_i,y_j)$. Then Eq. (4) can be reposed as

$$\begin{aligned} 4 \approx C(x,y)+\iint \limits_{\Omega _{x,y}} \frac{2 C(x^{\prime},y^{\prime})}{\pi }\arcsin \frac{1}{|\textbf{x}-\textbf{x}^{\prime}|}\frac{\,\text{d}\Omega }{d^2}, \end{aligned}$$

where

$$\begin{aligned} \Omega =[-(N+1/2)d,(N+1/2)d]\times [-(N+1/2)d,(N+1/2)d], \end{aligned}$$

is the full domain, and

$$\begin{aligned} \Omega _{x,y}=\Omega \setminus [(x-1/2)d,(x+1/2)d]\times [(y-1/2)d,(y+1/2)d], \end{aligned}$$

is the full geometry with a $d\times d$ square centred at (*x*, *y*) removed.

**Figure 5:**
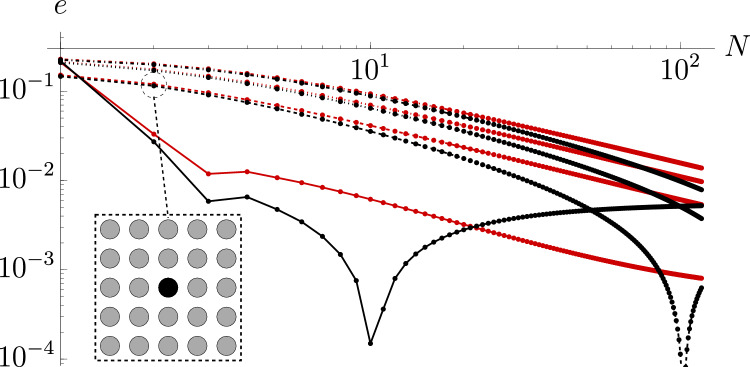
Error for Eq. (14) with $k=k_2$ (black) and $k=k_c$ (red) for $d=2$ (solid lines), $d=3$ (dashed lines), $d=4$ (dotted lines), $d=5$ (dot-dashed lines). Dots indicate integer values at which the error was calculated; these are connected by linear interpolants. The inset shows the array configuration for $N=2$, $d=3$.

For large *d*, this can be approached via a standard perturbation method in which $C(x,y)=4+O(d^{-1})$, although very large values of *d*, and/or multiple terms in the expansion (which are rather complicated due to reciprocal interactions) are required due to the simplistic nature of the leading-order approximation.

An alternate approach is to re-write the governing equation as

12 $$\begin{aligned} 4\approx \iint \limits _\Omega \frac{2C(x^{\prime},y^{\prime})}{\pi d^2}\arcsin \frac{1}{\max \{ 1,|\textbf{x}-\textbf{x}^{\prime}| \}}\,\text{d}\Omega . \end{aligned}$$

Note that $\arcsin (|\textbf{x}-\textbf{x}^{\prime}|^{-1})=|\textbf{x}-\textbf{x}^{\prime}|^{-1}+O\left( |\textbf{x}-\textbf{x}^{\prime}|^{-3}\right)$ and hence, as $|\textbf{x}-\textbf{x}^{\prime}|\ge 2d$ for $\textbf{x}^{\prime}\in \Omega _{x,y}$, this closely approaches the Green’s function formulation inverted to find Eq. (3) other than a small contribution close to (*x*, *y*). It is therefore suitable to approximate the capacity as $C(x,y)\approx C(0,0)\sigma (x,y)/\sigma (0,0)$, where $\sigma (x,y)$ is the density for a source with the shape of the array (here a square with side of length $(2N+1)d$). Evaluating at $(x,y)=(0,0)$,

13 $$\begin{aligned} 4&\approx C_{0,0}\left[ 1+\frac{2}{\pi d^2}\iint \limits_{\Omega _{0,0}} \frac{1}{r^{\prime}}\frac{\sigma (r^{\prime},\theta ^{\prime})r^{\prime}\,\text{d}r^{\prime} \,\text{d}\theta ^{\prime}}{\sigma (0,\theta ^{\prime})} \right] . \end{aligned}$$

Then

14 $$\begin{aligned} C_{0,0}\approx \frac{4}{1+\frac{2}{\pi d^2}(-4 d {{\,\text{arccoth}\,}}\sqrt{2} +\sigma _\text{int})}, \end{aligned}$$

where $\sigma _\text{int}=\iint _{\Omega } \sigma (r^{\prime},\theta ^{\prime})\,\text{d}r^{\prime} \,\text{d}\theta ^{\prime}/\sigma (0,\theta ^{\prime})=d(1/2+N)k$ and *k* is a parameter-free constant. We note that evaporation rates of other pixels can be approximated similarly, but we focus on (0, 0) as it is typically the rate-limiting contribution.

It is anticipated that the above approximation will give significantly better agreement with the exact solution than, for example, the expansion in large *d* due to the principal error arising from the approximation of the $\arcsin$ term which, as shown above, should be small.

The parameter *k* can be determined in a variety of ways. Using the leading order of Eq. (3) yields $k=4\pi {{\,\text{arccoth}\,}}\sqrt{2}\approx 11.08:=k_0$, while including second-order terms in $\epsilon$ yields $k\approx 11.17:=k_2$. A full calculation in COMSOL yields $k\approx 11.24:=k_c$.

We compare the results of Eqs. (4) to (14) in Fig. 5. This demonstrates excellent agreement: by $N=10^2$ all solutions have an error of $\lesssim \,1\%$. For $d\ge 3$ and $k=k_c$ the results exhibit power law behaviour with a slope of $-\,0.990$ indicating convergence for large *N*. Indeed, for large *N*, $C_{0,0}\sim 2d\pi /(kN)$, so for the approximate solution $k=k_2$ the lower limit on the error will be $\approx \,0.6\%$. The non-monotonic behaviour for $k=k_2$ is due to the solutions for $d=2$ and $d=3$ switching from underestimating to overestimating at $N=11$ and $N=103$ respectively, before converging to 0.6% for large *N*.

Note that the range of validation of the approximate solution Eq. (14) in Fig. 5 is restricted by memory constraints: when solving the full system Eq. (4) with 32 gigabytes of memory at 16 bytes per matrix entry, the maximum theoretical dense matrix size is $M \times M$ with $M=(32 \times 1024^3/16)^{1/2}\approx 4.6 \times 10^4$ droplets (corresponding to $N=107$). This is still 4 orders of magnitude short of the scale required for ultra high definition screens, effectively enforcing the use of a methodology such as that presented here. Indeed, to that end, calculating Eq. (14) is practically instantaneous, so that the favourable comparisons presented herein demonstrate that our substantially simpler and quicker approach provides an excellent methodology with potential in future applications involving large arrays of sources.

### Densities of multiple sources

Finally, we wish to examine the most stringent possible test for our formulation. As observed by Ref.^5^, the most stringent test of any approximate or asymptotic solution of Eq. (1) should be given by several sources with domains of similar area in close proximity.

**Figure 6:**
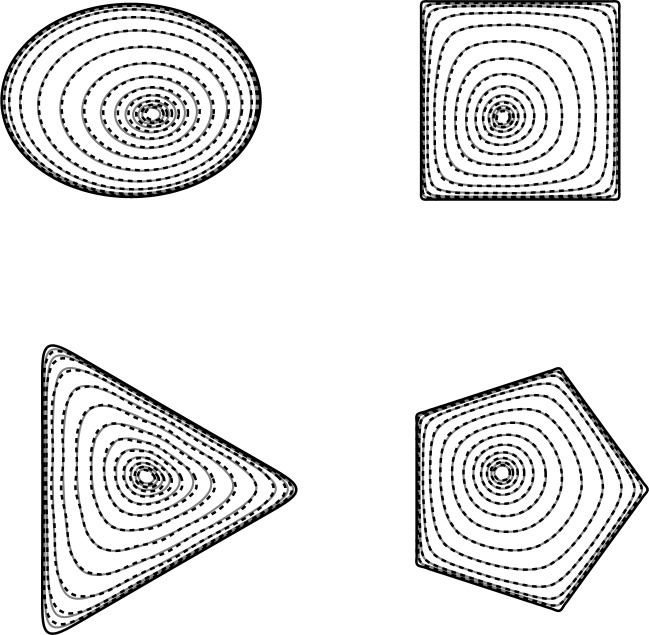
Contours of local heat flux calculated using Eq. (3) (dashed lines) and COMSOL (solid lines) for an array of elliptic ($g=0.238$; 0.727% error), square ($g=0.244$; 0.161% error), triangular ($g=0.256$; 3.60% error) and pentagonal ($g=0.239$; 0.878% error) plates held at uniform temperature.

Following the formulation of Ref.^1,2^, the heat flux from a plate held at constant temperature corresponds to the density of a source at a constant potential. Therefore, for an array of several plates at constant temperature, the solutions for the heat flux are given by the results of Eqs. (3) and (4); these are compared to those of COMSOL in Fig. 6. For each plate, the predictions of both local heat flux, as illustrated by the contours, and net heat flux, measured by *g*, are in excellent with the COMSOL solutions. This is encouraging, and indicates that the model Eqs. (3) and (4) is robust and suitable for use in challenging contexts.

Several features are of note in this figure. Firstly, comparison of the *g* values against those given in Fig. 3 immediately indicates a decrease in the total capacity of each source, corresponding to a decrease in total heat flux. This is due to the interference of the other plates: a phenomenon known in evaporative contexts as shielding^22^. Essentially, the heat due to the other plates increases the local temperature, decreasing the thermal gradient, and hence decreasing the thermal flux.

Secondly, all the minima (i.e. the positions of minimal thermal flux) are displaced from the centre of the respective plates. This is again due to the shielding effect: where the other plates are closer, the effect is more pronounced, resulting in a shift of the minimum. The combined effect of multiple other plates tends to result in a shift towards the “centre” of the other three: the minimum for the ellipse in the top left is shifted towards the bottom right, as all the other plates are to the right of and/or below the ellipse.

Thirdly, the shape of the contours close to the minimum is no longer as simple as was observed for single plates in the “Density of an isolated source” section. In particular, the triangle, which has no $n=2$ mode and thus has circular contours close to the minimum in isolation, is distorted to a shape close to an ellipse. This is due to the complexity of the interference of the other plates.

Finally, we note that nonetheless a qualitatively similar behaviour is observed to the single-source case: close to the boundary, the contours are close to scaled copies of the boundary, but these smooth towards the minimum.

Obviously, there is much scope to explore such an incredibly diverse problem as Eq. (1), with a great deal more behaviours to consider, but they are beyond the scope of the present article in which we have established a reliable, fast asymptotic solution to the mixed boundary value problem.

## Discussion

Herein, we have presented a robust and accurate explicit solution to the the classical potential problem Eq. (1) given by Eqs. (3) and (4). While the solution is formally only valid in the asymptotic limit in which the sources are nearly-circular, we have demonstrated for a range of different test cases that it gives excellent results even for sharp polygons and highly non-convex shapes. Given the ubiquity of Eq. (1) in different physical systems, ranging from thermostatics to porous layers to ice crystal growth, our solution represents a significant step forward to approaching these notoriously complex mathematical problems.

In particular, we have examined systems of interest in electrostatics, droplet evaporation and thermostatics, in each case showing excellent agreement with both experimental data and COMSOL simulations. In the latter, it is notable that these simulations are expensive and unwieldy, finding significant difficulties in resolving the singular behaviour near the change in boundary condition. These complexities scale up rapidly as the number of sources increases. Our solution offers an accurate, reliable, and rapid alternative approach.

Finally, motivated by printing pixels in OLED screen manufacture, we introduced a rapid solution methodology for problems involving large arrays of sources, where the quantity of industrial relevance is the total evaporation rate (i.e. the capacity). By noting similarities to the Green’s function formulation of the classical potential problem, we were able to reduce calculating the capacity to simple quadrature based on our asymptotic solution. We demonstrated excellent agreement between our approach and full numerical simulations.

We have shown that the methodology described herein has already opened an array of new problems to analytic tractability, including non-circular electrodes, and the evaporation of large arrays of droplets as seen in screen printing problems. Further problems in this direction, especially analytic treatment of the deposition from non-circular droplets and the effect of general arrays of stamps on linear elastic media, will form the focus of future investigations.

### Supplementary Information

Supplementary Information.

## Data Availability

The datasets used and/or analysed during the current study available from the corresponding author on reasonable request.
