# Supplementary material for: High-order asymptotic methods provide accurate, analytic solutions to intractable potential problems
Source: Sci Rep. 2024 Feb 20;14:4225. doi: 10.1038/s41598-024-54377-2 (PMC10879137; doi:10.1038/s41598-024-54377-2)
Supplement: Supplementary file 1 — Supplementary Information. [file 41598_2024_54377_MOESM1_ESM.pdf]

Supplementary information

Alexander W. Wray*
Department of Mathematics and Statistics, University of Strathclyde, Livingstone Tower, 26 Richmond Street, Glasgow G1 1XH, UK

Madeleine R Moore†
Department of Mathematics, School of Natural Sciences, University of Hull, Cottingham Road, Hull, HU6 7RX, UK
(Dated: February 1, 2024)

ISOLATED SOURCE

We first consider the case of an isolated source. A standard Green's function formulation of Eq. (1) in the main document yields

$$c_S(r,\theta) = \frac{1}{2\pi}\int_{\theta'=0}^{2\pi}\int_{r'=0}^{a(\theta')}\frac{\sigma(r',\theta')r'\,\mathrm{d}r'\,\mathrm{d}\theta'}{\sqrt{r'^2+r^2-2r'r\cos(\theta-\theta')}} \tag{1}$$

for $r < a(\theta)$, where $a(\theta) = 1 + \epsilon f(\theta)$, and f can be decomposed into a suitable finite Fourier series as

$$f(\theta) = \sum_{i=2}^{M} B_i(\theta) \quad \text{where} \quad B_i(\theta) = a_i\cos(i\theta) + b_i\sin(i\theta), \tag{2}$$

where $M \geq 2$. We note that the series may be chosen to start at $i = 2$ without loss of generality by selecting a suitable centre for the coordinate system for the source, as seen in Wray and Moore [1].

Then using a decomposition due to Copson [2] and Fabrikant [3], this can be written as

$$c_S(r,\theta_1) = \frac{1}{\pi^2}\int_0^r \frac{\mathrm{d}x}{(r^2-x^2)^{1/2}}\int_0^{2\pi}\mathrm{d}\theta'\int_x^{a(\theta')}\frac{r'\,\mathrm{d}r'}{(r'^2-x^2)^{1/2}}\mathcal{L}\left(\frac{x^2}{r'r},\theta_1-\theta'\right)\sigma(r',\theta'), \tag{3}$$

where the $\mathcal{L}$ operator, as described by Fabrikant [3], is given by

$$\mathcal{L}(k,\theta) = 1 + 2\sum_{j=1}^{\infty}k^j\cos j\theta \approx 1 + 2\sum_{j=1}^{K}k^j\cos j\theta, \tag{4}$$

where for our purposes it suffices to truncate at $K = 2M$. Note that, in Eq. (3), we have introduced the variable θ_1 for convenience in the inversions that follow: it is simply the standard planar polar angle.

The key property to be used repeatedly is that

$$\int_0^{2\pi}\mathcal{L}(k,\phi-\phi_1)\mathcal{L}(k_1,\phi_1-\psi)\,\mathrm{d}\phi_1 = 2\pi\mathcal{L}(kk_1,\phi-\psi). \tag{5}$$

Throughout, $\mathcal{L}$-operators will be inverted using Eq. (5), while Abel-type operators will be inverted in the standard manner of Copson [2]. The expression given in Eq. (3) is the integral equation to be inverted for σ. We now demonstrate how to do this for the case $c_S \equiv 1$, although other choices proceed in a similar manner. Inverting the outermost $\mathcal{L}$ and Abel operators yields

$$2\pi = \int_0^{2\pi}\mathrm{d}\theta'\int_x^{a(\theta')}\frac{r'\,\mathrm{d}r'}{(r'^2-x^2)^{1/2}}\mathcal{L}\left(\frac{x^2}{r'},\theta_1-\theta'\right)\sigma(r',\theta'). \tag{6}$$

Applying

$$\int_0^{2\pi}\mathcal{L}(x^{-2},\theta-\theta_1)\cdot\,\mathrm{d}\theta_1 \tag{7}$$

and making use of (5) yields

$$2\pi = \int_0^{2\pi} \mathrm{d}\theta' \int_x^{a(\theta')} \frac{r'\,\mathrm{d}r'}{(r'^2 - x^2)^{1/2}} \mathcal{L}\left(\frac{1}{r'}, \theta - \theta'\right) \sigma(r', \theta'). \tag{8}$$

This is now solved order by order by expanding

$$\sigma = \sigma_0 + \epsilon\sigma_1 + \epsilon^2\sigma_2 + \mathcal{O}\left(\epsilon^3\right) \quad \text{as} \quad \epsilon \to 0. \tag{9}$$

Note that the solutions for each of these are given below explicitly in Eq. (11), Eq. (20) and Eq. (30) respectively.

Leading order

At leading order, the solution trivially coincides with that of a circular disk at uniform potential,

$$\sigma_0(r, \theta) = \frac{2}{\pi} \frac{1}{\sqrt{1 - r^2}}, \tag{10}$$

as first demonstrated by [4]. In order to correctly locate the square root singularity at the contact line while retaining smoothness at the origin, we use the asymptotically equivalent solution

$$\sigma_0 = \frac{2}{\pi} \frac{a(\theta)}{\sqrt{a(\theta)^2 - r^2}}. \tag{11}$$

First order

At first order, Eq. (8) yields

$$2\pi = \int_0^{2\pi} \mathrm{d}\theta' \int_x^{a(\theta')} \frac{r'\,\mathrm{d}r'}{(r'^2 - x^2)^{1/2}} \mathcal{L}\left(\frac{1}{r'}, \theta - \theta'\right) \sigma_0(r', \theta') \tag{12}$$

$$+ \epsilon \int_0^{2\pi} \mathrm{d}\theta' \int_x^{1} \frac{r'\,\mathrm{d}r'}{(r'^2 - x^2)^{1/2}} \mathcal{L}\left(\frac{1}{r'}, \theta - \theta'\right) \sigma_1(r', \theta'). \tag{13}$$

The terms on the first line can be thought of as forcing terms for the standard Abel-type problem on the second line. We therefore compute

$$\mathcal{F}_1 = \int_0^{2\pi} \mathrm{d}\theta' \int_x^{a(\theta')} \frac{r'\,\mathrm{d}r'}{(r'^2 - x^2)^{1/2}} \mathcal{L}\left(\frac{1}{r'}, \theta - \theta'\right) \frac{2}{\pi} \frac{a}{\sqrt{a^2 - r'^2}} \tag{14}$$

$$= 2\pi \int_x^{a(\theta')} \frac{r'\,\mathrm{d}r'}{(r'^2 - x^2)^{1/2}} \frac{2}{\pi} \frac{a}{\sqrt{a^2 - r'^2}} + 2\sum_{j=1}^{K} \int_0^{2\pi} \mathrm{d}\theta' \cos j(\theta - \theta') \int_x^{a(\theta')} \frac{r'^{1-j}\,\mathrm{d}r'}{(r'^2 - x^2)^{1/2}} \frac{2}{\pi} \frac{a}{\sqrt{a^2 - r'^2}}.$$

Making use of the helpful result

$$\int_x^a \frac{(r')^{1-m}}{\sqrt{(r')^2 - x^2}\sqrt{a^2 - (r')^2}} \,\mathrm{d}r' = \frac{\pi}{2a}\, {}_2F_1\left(1 - \frac{m}{2}, \frac{1}{2}; 1; 1 - \frac{x^2}{a^2}\right) x^{1-m} \tag{15}$$

yields

$$\mathcal{F}_1 = 2\pi + 2\sum_{j=1}^{K} \int_0^{2\pi} \mathrm{d}\theta' \cos j(\theta - \theta') \left[c(x) - \frac{1}{2}\epsilon f(\theta')(j-2)x^2\, {}_2F_1\left(\frac{3}{2}, 2 - \frac{j}{2}; 2; 1 - x^2\right)\right] x^{1-j}, \tag{16}$$

where $c(x) = {}_2F_1\left(\frac{1}{2}, 1 - \frac{j}{2}; 1; 1 - x^2\right)$ is independent of θ' and so does not contribute to the integral. Then

$$\mathcal{F}_1 = 2\pi - \epsilon\pi \sum_{j=2}^{K} B_j(\theta)(j-2)x^{3-j}\, {}_2F_1\left(\frac{3}{2}, 2 - \frac{j}{2}; 2; 1 - x^2\right), \tag{17}$$

where we have made use the Fourier series expansion for $f(\theta)$. Thus Eq. (13) yields

$$\pi \sum_{j=2}^{K} B_j(\theta)(j-2)x^{3-j}\, {}_2F_1\left(\frac{3}{2}, 2-\frac{j}{2}; 2; 1-x^2\right) = \int_x^1 \frac{\mathrm{d}r'}{(r'^2-x^2)^{1/2}} \int_0^{2\pi} \mathcal{L}\left(r'^{-1}, \theta-\theta'\right) r'\sigma_1(r',\theta')\,\mathrm{d}\theta',$$

which can be inverted to find

$$\sigma_1(r,\theta) = -\frac{1}{\pi}\sum_{j=2}^{K} B_j(\theta)(j-2)r^{j-1}\frac{\mathrm{d}}{\mathrm{d}r}\int_r^1 \frac{t^{4-j}}{(t^2-r^2)^{1/2}}\, {}_2F_1\left(\frac{3}{2}, 2-\frac{j}{2}; 2; 1-t^2\right)\mathrm{d}t \tag{18}$$

$$= -\frac{1}{\pi}\sum_{j=1}^{K} B_j(\theta)(j-2)r^{j-1}\frac{2r(1-r^{2-j})}{(j-2)(1-r^2)^{3/2}}. \tag{19}$$

Again, we may re-write this in a suitable asymptotically equivalent form,

$$\sigma_1 = \sum_{j=2}^{K} B_j(\theta)\frac{2}{\pi}\frac{r^2}{a^2}\frac{(1-(r/a)^{j-2})}{(1-(r/a)^2)^{3/2}}. \tag{20}$$

Second order

The forcing terms up to second order are

$$\mathcal{F}_2 = \int_0^{2\pi} \mathrm{d}\theta' \left[1 + 2\sum_{j=1}^{K}\cos j(\theta-\theta')\right] \times I_j(\theta', x), \tag{21}$$

where

$$I_j(\theta',x) = \int_x^{a(\theta')} \frac{r'^{1-j}\,\mathrm{d}r'}{(r'^2-x^2)^{1/2}}\frac{2}{\pi}\frac{a}{\sqrt{a^2-r'^2}}\left[1+\epsilon\sum_{n=2}^{K} B_n(\theta')\frac{r'^2}{a^{n-2}}\frac{a^{n-2}-r'^{n-2}}{a^2-r'^2}\right],$$
$$= x^{1-j}\Bigg\{{}_2F_1\left(\frac{1}{2}, 1-\frac{j}{2}; 1; 1-\frac{x^2}{a^2}\right) +$$
$$\epsilon\Bigg(\sum_{n=2}^{K}\frac{1}{2}B_n\bigg[-\left((j-n)\left(\frac{x}{a}\right)^n {}_2F_1\left(\frac{3}{2}, \frac{1}{2}(-j+n+2); 2; 1-\frac{x^2}{a^2}\right)\right)$$
$$-2\,{}_2F_1\left(\frac{1}{2}, 1-\frac{j}{2}; 1; 1-\frac{x^2}{a^2}\right) + j\,{}_2F_1\left(\frac{3}{2}, 1-\frac{j}{2}; 2; 1-\frac{x^2}{a^2}\right)\bigg]\Bigg)\Bigg\}. \tag{22}$$

Then, we may expand

$$I = I_{j,0} + \epsilon I_{j,1} + \epsilon^2 I_{j,2} + O(\epsilon^3) \tag{23}$$

as $\epsilon \to 0$, where we find that

$$I_{j,0} = x^{1-j}\,{}_2F_1\left(\frac{1}{2}, 1-\frac{j}{2}; 1; 1-x^2\right) \tag{24}$$

$$I_{j,1} = \frac{1}{2}x^{1-j}\left(\frac{2x^2(\sum_m B_m(\theta') - \sum_n B_n(\theta'))\left({}_2F_1\left(\frac{1}{2}, 1-\frac{j}{2}; 1; 1-x^2\right) - {}_2F_1\left(\frac{3}{2}, 1-\frac{j}{2}; 1; 1-x^2\right)\right)}{x^2-1}\right.$$
$$\left. - \sum_n B_n(\theta')(j-n)x^n\,{}_2F_1\left(\frac{3}{2}, \frac{1}{2}(-j+n+2); 2; 1-x^2\right)\right), \tag{25}$$

$$I_{j,2} = \frac{3}{16}(j-2)\left(\sum_m B_m(\theta')\right)^2\left[4\,{}_2F_1\left(\frac{1}{2}, \frac{j}{2}; 2; 1-x^2\right) + (j-4)\,{}_2F_1\left(\frac{1}{2}, \frac{j}{2}; 3; 1-x^2\right)\right]$$
$$- \frac{1}{8}(j-2)\sum_n\sum_m B_m(\theta')B_n(\theta')\left[3j\,{}_2F_1\left(\frac{1}{2}, \frac{j}{2}+1; 3; 1-x^2\right) - 4\,{}_2F_1\left(\frac{1}{2}, \frac{j}{2}; 2; 1-x^2\right)\right]$$
$$+ \frac{1}{8}\left(\sum_m B_m(\theta')\right)\sum_n B_n(\theta')(j-n)\left[4n\,{}_2F_1\left(\frac{1}{2}, \frac{1}{2}(j-n+2); 2; 1-x^2\right)\right.$$
$$\left. +3(j-n-2)\,{}_2F_1\left(\frac{1}{2}, \frac{1}{2}(j-n+2); 3; 1-x^2\right)\right]. \tag{26}$$

At leading and first order all terms cancel, verifying Eq. (11) and Eq. (20), and hence the remaining system to solve is

$$\int_0^{2\pi} \mathrm{d}\theta'\left[I_{j,2} + \int_x^1 \frac{r'\,\mathrm{d}r'}{(r'^2-x^2)^{1/2}}\mathcal{L}\left(\frac{1}{r'}, \theta-\theta'\right)\sigma_2\right] = 0. \tag{27}$$

Inverting the Abel operator yields

$$\int_0^{2\pi} r'\mathcal{L}\left(r'^{-1}, \theta-\theta'\right)\sigma_2\left(r', \theta'\right)\,\mathrm{d}\theta' = \frac{2}{\pi}\frac{\mathrm{d}}{\mathrm{d}r'}\int_{r'}^1 \frac{t\,\mathrm{d}t}{(t^2-r'^2)^{1/2}}\int_0^{2\pi}\mathcal{L}(1, \theta-\theta')I_{j,2}\,\mathrm{d}\theta'. \tag{28}$$

Then,

$$\int_0^{2\pi} r'\mathcal{L}\left(r^{-1}, \theta-\theta'\right)\sigma_2\left(r, \theta'\right)\,\mathrm{d}\theta' = \frac{2}{\pi}\int_0^{2\pi}\mathcal{L}(1, \theta-\theta')\times$$
$$\left[\left(\sum_m B_m(\theta')\right)^2 \frac{r\left(-3j\left(r^2-1\right) - 2\left(2r^2+1\right)r^{2-j} + 10r^2 - 4\right)}{4\left(1-r^2\right)^{5/2}}\right.$$
$$\left. + \left(\sum_m B_m(\theta')\right)\sum_n B_n(\theta')\frac{r\left(-r^2(-3j+n+6) + \left(2n - 2(n-3)r^2\right)r^{n-j} - 3j + n\right)}{2\left(1-r^2\right)^{5/2}}\right]\mathrm{d}\theta', \tag{29}$$

and hence

$$\sigma_2(r, \theta) = \frac{1}{\pi^2}\left[r^{-1}c_0(r) + \sum_{j=1}^K r^{j-1}(c_j(r)\cos j\theta + d_j(r)\sin j\theta)\right], \tag{30}$$

where the $c_j(r)$ and $d_j(r)$ are the Fourier coefficients corresponding to the terms in square brackets as given by Eq. (29). Note that this recovers the monochromatic solution given by Wray and Moore [1] in the appropriate case, but accurately treats the second order terms which were dealt with approximately therein. In line with Eq. (11) and Eq. (20), we map r to $r/a(\theta)$ in practical applications to ensure that singularities remain at the contact line while preserving appropriate smoothness properties at $r = 0$.

For the large values of j and ϵ encountered in extending outside the asymptotic limit to accommodate shapes such as polygons, Eq. (30) can occasionally yield unphysical divergence to negative infinity [1]. Experimentation suggests that when determining the density for shapes with Fourier expansions that correspond to large values of ϵ (i.e. ones that fall far outside the asymptotic limit, such as those found for polygons), it is in fact best to truncate the sum Eq. (30) at twice the rotational symmetry of the source boundary.

MULTIPLE SOURCES

Consider a system of N sources as shown in Figure 1 of the main document. The equivalent Green's function formulation to Eq. (1) for source S_i is now

$$1 = \sum_k \frac{1}{2\pi} \iint_{S_k} \frac{\sigma_k(r',\theta')r'\,\mathrm{d}r'\,\mathrm{d}\theta'}{\sqrt{r'^2 + r^2 - 2r'r\cos(\theta-\theta')}} \tag{31}$$

for $(r',\theta') \in S$ and $(r,\theta) \in S_i$. Setting

$$\sigma_i = \sigma_i^I + \sigma_i^B, \tag{32}$$

where σ_i^I is the solution for a source in isolation as given by Eq. (9), Eq. (11), Eq. (20), Eq. (30), and σ_i^B is the perturbation due to being in proximity to other sources, Eq. (31) becomes

$$\begin{aligned} 0 =& \frac{1}{\pi^2}\int_0^r \frac{\mathrm{d}x}{\sqrt{r^2-x^2}} \int_x^{a_1(\theta)} \frac{r'\,\mathrm{d}r'}{\sqrt{r'^2-x^2}} \int_0^{2\pi} L\left(\frac{x^2}{rr'},\theta-\theta'\right)\sigma_1^B(r',\theta')\,\mathrm{d}\theta' \\ &+ \frac{1}{\pi^2}\sum_{k=2}^N \int_0^r \frac{\mathrm{d}x}{\sqrt{r^2-x^2}} \iint_{S_n} \frac{r'\,\mathrm{d}r'}{\sqrt{r'^2-x^2}} L\left(\frac{x^2}{rr'},\theta-\theta'\right)\sigma_k(r',\theta')\,\mathrm{d}\theta', \end{aligned} \tag{33}$$

for $i = 1$. We assume that the sources are well-separated, so that we may introduce the small parameter

$$\delta = \max_{k,n;k\neq n} \frac{\bar{a}_k}{r_{k,n}} \ll 1. \tag{34}$$

To simplify inversion, we change the upper limit of the second integral in the first line of Eq. 33 to $\bar{a}_1$, incurring an error of $O(\epsilon\delta)$. Then the formulation of Fabrikant [3] may be followed directly to yield

$$\sigma_1^B(r,\theta) = -\frac{1}{\pi^2\sqrt{\bar{a}_1^2 - r^2}} \sum_{n=2}^N \iint_{S_n} \frac{\sqrt{r'^2-\bar{a}_1^2}\,r'\,\mathrm{d}r'}{r^2 + r'^2 - 2rr'\cos(\theta-\theta')}\sigma_n(r',\theta')\,\mathrm{d}\theta'. \tag{35}$$

Finally, as shown by Wray et al. [5], we may take $r' \approx r_{i,j}$ and $\theta' \approx \theta_{i,j}$, where the error incurred is of $O(\delta^2)$. We may therefore write

$$\sigma_1^B(r,\theta) = -\frac{1}{2\pi}\sigma_1^I \sum_{n=2}^N \frac{\sqrt{r_{1,n}^2 - a_{1,0}^2}}{r^2 + r_{1,n}^2 - 2rr_{1,n}\cos(\theta-\theta_{1,n})}\frac{C_n}{\bar{a}_1}, \tag{36}$$

where the C_n may be determined to high accuracy in the usual way via the reciprocal method (see, for example, Fabrikant [6]). Hence

$$\sigma_i = \sigma_i^I\left(1 - \sum_{n\neq i}^N S(r,\theta;i,n)\right), \tag{37}$$

where

$$S(r,\theta;i,n) = \frac{1}{2\pi}\frac{\sqrt{r_{i,n}^2 - \bar{a}_i^2}}{r^2 + r_{i,n}^2 - 2rr_{i,n}\cos(\theta-\theta_{i,n})}\frac{C_n}{\bar{a}_i} \tag{38}$$

is the shielding of source i due to source n.

* alexander.wray@strath.ac.uk
† m.r.moore@hull.ac.uk
[1] A. W. Wray and M. R. Moore, J. Fluid Mech. **961**, A11 (2023).
[2] E. Copson, Proc. Edin. Math. Soc. **8**, 14 (1947).
[3] V. Fabrikant, ZAMP **36**, 616 (1985).
[4] H. Weber, J. für die reine Angew. Math. **75**, 75 (1873).
[5] A. W. Wray, B. R. Duffy, and S. K. Wilson, J. Fluid Mech. **884**, A45 (2020).
[6] V. Fabrikant, J. Appl. Phys. **61**, 813 (1987).
